# Tracing recombinant bovine somatotropin ab(use) through transcriptomics: the potential of bovine somatic cells in a multi-dose longitudinal study

**DOI:** 10.1038/s41598-019-41343-6

**Published:** 2019-03-18

**Authors:** Alexandre Lamas, Patricia Regal, Beatriz Vázquez, José Manuel Miranda, Alberto Cepeda, Carlos Manuel Franco

**Affiliations:** Laboratorio de Higiene Inspección y Control de Alimentos. Departamento de Química Analítica, Nutrición y Bromatología. Universidade de Santiago de Compostela, 27002 Lugo, Spain

## Abstract

In the European Union, the use of recombinant bovine somatotropin (rbST) in dairy cattle is forbidden. Monitoring rbST (ab)use by its direct detection in animal matrices still remains a challenging task. New monitoring methods based on indirect detection of the substance are necessary. A new transcriptomic system based on the use of high-throughput real-time PCR in combination with somatic cells was developed to control rbST administration in dairy animals. A total of nine cows, separated into control and rbST-treated groups, were included in the study. A subcutaneous injection containing 500 mg of rbST was administered to the treated group every 14 days, up to a total of 12 doses. Milk somatic cells (MSCs) were sampled from each animal at different time points throughout 8 months of study. It was possible to obtain the transcriptomic profile of 18 genes in MSCs of rbST-treated and control groups, and using univariate and multivariate statistical analysis control and treated animals were discriminated. The transcription of CCND1, IGF-1R, TNF and IL-1β genes resulted strongly influenced by rbST treatment. The combination of MSCs, transcriptomic tools and statistical analysis has allowed the selection of four genes as potential biomarkers that could be used in a transcriptomic panel for monitoring rbST administration in cows.

## Introduction

Bovine somatotropin (bST) is a peptide hormone synthesized by cows’ pituitary glands. It acts by binding to membrane-bound receptors located in different tissues such as liver, bone or mammary glands. A pioneer study by Asimov and Krouze^1^ in the 1930s discovered that injections of pituitary extract induced an increase in milk yield of dairy cattle, and research carried out by Folley and Young found that these galactopoietic effects were due to somatotropin^2,3^. The development of DNA recombinant technology made the industrial production of recombinant bovine somatotropin (rbST) economically practicable^4^. This production system opened the door for the commercial use of rbST in dairy cows to increase milk yield. In 1993, the Food and Drug Administration of the United States (FDA Agency) approved for the first time the use of rbST in dairy cows. Although possible rbST residues contained in milk from treated animals should be destroyed in the human digestive system, there are some non-clarified concerns about its safety for consumers. As a matter of fact, it has been reported that rbST treatment may cause an increase on insulin-like growth factor (IGF-1) in milk, in comparison to milk from non-treated dairy cattle^5–7^. As IGF-1 levels are not altered by pasteurization of milk^8^, the hypothesis of IFG-1 reaching the consumer seems plausible and biologically feasible^9^.

The use of rbST in cattle is legal in some countries such as the United States, Mexico and Brazil. However, in 1999, the European Union decided to ban the use of rbST invoking animal welfare reasons and the impact of European milk policy and consumers fears^10^. The banning of rbST resulted in the need for developing analytical methods to detect its fraudulent use. As a relatively recent example, in 2013 the Spanish authorities detected that many farmers had been administering rbST to their cows using commercial injections of rbST (Lactotropin^®^ and Boostin^®^) from Mexico illegally introduced into Spain. In a recent report, Sterk^11^ established the use of rbST as a current challenge to European residue control plans. In this context, analytical methods such as liquid chromatography coupled to mass spectrometry, based on the direct detection of the banned substance, are the first option to detect its use^12^. In this context, some research groups have successfully developed some methods based in the use of liquid chromatography coupled to mass spectrometry to detect rbST in plasma, serum^13–16^ and milk^17^. However, some commercially available forms rbST have the same amino acid composition as natural bST, rendering practically impossible their differentiation^18^. Therefore, it is also of great importance to develop indirect methods that allow detection of rbST administration in cattle. In this sense, some research works have developed ELISA methods to detect rbST antibodies in serum and milk^19,20^. Other studies have developed methods based in the determination of multiple protein biomarkers to detect the use of rbST in dairy cattle^5,21^. Even, the clinical biochemical and hormonal profiling in plasma was evaluated as a potential tool to predict the use of rbST in cattle^22^.

In recent years, transcriptomics technology has experienced a boom due to the development of RNA sequencing (RNA-seq), microarrays and high-throughput real-time PCR systems. While RNA-seq and microarrays perform massive measurements of the transcriptome in a reduced number of samples, high-throughput real-time PCR enables the analysis of various genes at the same time in a large number of samples^23^. Besides, real-time PCR is considered the gold standard for quantification purposes, permitting identification of small differences between samples with better precision than RNA-seq and microarrays. Recently, some research groups have used transcriptomics as a tool to detect the use of growth promoters in beef cattle^24,25^. However, these studies focused on the use of target tissues such as liver or muscle obtained after slaughtering. Other studies have been focused on the use of blood cells^26^. Although blood samples enable transcriptomic controls *in vivo*, they require direct contact with animals and invasive sampling procedures. In the specific case of rbST, it is essential to monitor its misuse during the period of lactation, before milk reaches the market, and therefore the collection of samples after slaughtering animals is not a real option in terms of food safety. All this means that target samples must be easy to collect, the method of sample collection must cause minimal or no pain to the animal, and it has to be economically viable. One of the main target tissues of rbST in dairy cattle is the mammary gland, where this peptide hormone exerts an important galactopoietic effect. Due to this direct relationship, recent studies have used post-mortem mammary tissue to evaluate the effect of rbST on its transcriptional profile^27^. However, as mentioned before, it is important to detect the use of this substance during lactation to avoid the entry of milk produced using rbST into the market. At this point, milk somatic cells appear as a good alternative to carry out *in vivo* transcriptomic assays to detect the use of rbST. A comparative study, in which five different RNA sources were evaluated to examine the lactating bovine mammary gland transcriptome, concluded that detecting MSCs released into milk during lactation is one of the simplest methods to isolate RNA. Also, the MSC transcriptome is representative of mammary gland tissue and can be used as an effective alternative to study mammary gland tissue gene expression without the need for a tissue biopsy^28^. In a practical way, Toral *et al*.^29^ demonstrated the potential of MSCs from dairy ewes as an alternative to mammary biopsies in performing nutrigenomic studies.

The aim of this study is to evaluate the potential of high-throughput real-time PCR to obtain a gene expression profile in MSCs collected from cows treated with rbST. To represent real farm conditions and to differentiate this work from the transcriptomic studies carried out until now in relation to rbST, nine cows were housed separately in a semi-extensive dairy farm, and a total of 12 injections of Lactotropina® were administered to six of them, in two-week intervals. MSCs were collected from six treated cows and three control animals at 36 different time points to analyse the expression of selected genes and seek transcriptomic differences between groups. Additional control samples from rbST-free farms have been also included.

## Material and Methods

### Animals and treatments

**Figure 1:**
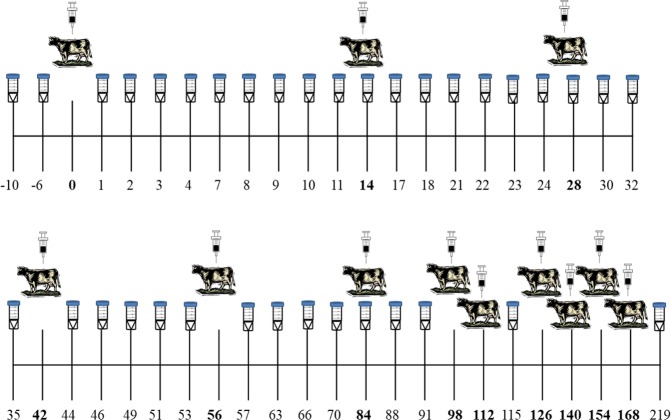
Overview of the rbST animal experiment, showing the days of milk sample collection (marked with a tube with a blue cap), and the days of animal treatment (days highlighted in bold numbers).

As described in a previous work carried out by this research group^30^, nine Holstein cows in first or second lactation and in an age range from 1.5 to 4 years were chosen from a herd of dairy cows and housed separately at the same farm. Feed was distributed to the cows twice a day, and they had *ad libitum* access to fresh water. Milking was carried out in a herringbone milking parlor in two sessions, at 8:00 and 18:00 hours. Milk production in volume of each individual animal was recorded every day during eight consecutive months (240 days) by using individual milk meters, starting collecting data eighteen days before the first rbST dose (pre-dose or conditioning period) and finalizing 1.8 months (54 days) after the last dose of the treated group. The nine cows were divided into two groups: control group composed of three cows and rbST-treated group composed of six cows. The rbST group was treated with 500 mg of rbST (Lactotropina^®^, Elanco^®^, Eli Lilly, Mexico) subcutaneously every 14 days (the period between a rbST injections and the next administration is considered a rbST cycle), according to the manufacturer’s recommendations, during a total period of 6 months. Lactotropina® syringes were kept refrigerated and left to warm to room temperature before use. During each treatment, a veterinary surgeon introduced the syringe subcutaneously behind the shoulder after removing surface dirt with alcohol and alternating between the cow’s left and right side on consecutive injections. At the time of the first dose, rbST animals have been 67 ± 4 days in lactation (lactation peak) and control animals 75 ± 4 days. A total of 12 rbST doses were administered to the treated group during the study, and milk samples of the first milking of the day were collected from both control ant treated animals at different time points during the whole experiment (Fig. 1). Between the fifth and sixth rbST treatment, there was a 28-day gap with no rbST dose. Also, since the last rbST dose was administered, milk production was recorded by two months. It was made to determine the existence of some effect of this lack of dose on gene expression of rbST group in comparison to control group. Experimental procedures were performed after evaluation and on approval of the corresponding regional authorities (Service of Livestock Farming of the Provincial Government of Lugo, Regional Ministry of Rural Affairs, Galicia), in accordance with EU guidelines and national laws on animal experiments, in particular, Directive 2010/63/EU on the protection of animals used for scientific purposes, and its transposition into national law. All procedures were performed respecting animal welfare and causing no more pain, suffering, distress or lasting harm than the equivalent to that caused by the introduction of a needle in accordance with good veterinary practice.

### Milk somatic cell collection

The collection of milk somatic cells (MSCs) was carried out as previously described by our research group^30^. Briefly, a total of two litres of homogenized milk from the morning milking of each cow were collected at the milking parlour in sterile bottles (Deltalab, Spain) and immediately transported in refrigerated conditions to the laboratory. Before milking, udder was cleaned and first contaminated milk streams were dismissed. The remaining milk that was not collected for experimental assays was discarded. A total volume of 225 mL of fresh milk was used to collect MSCs for analysis. For this purpose, 50 mL conical centrifuge polypropylene tubes were filled with 45 mL of milk and centrifuged at 2,300 RCF for 10 min at 4 °C. The supernatant (fat and whey) was discarded, keeping the pellet, and then the tubes were filled again with 45 mL of milk and centrifuged once more. This step was repeated five times for each sample in the same conical plastic tube to concentrate the pellet. Then, the milk pellet containing the MSCs was mixed with 1 mL of TRIzol (Ambion^TM^_,_ Thermo Fisher Scientific, Massachusetts, USA), transferred to a 1.5 mL microtube and stored at −20 °C until further use. Following the same procedure, a total of three MSCs samples from three different cows belonging to an external dairy farm unrelated to the farm where the cows of the study were housed were collected.

### Total RNA extraction and reverse transcription

As described in a previous work^30^, total RNA was extracted using TRIzol Reagent (Ambion^TM^, Thermo Fisher Scientific, Massachusetts, USA) according to the manufacturer’s protocol. Quantification of the RNA was carried out with a Qubit^®^ RNA BR Assay Kit and a Qubit^®^ fluorometer (Invitrogen^TM^, Thermo Fisher Scientific). The A260/280 ratio of RNA samples was determined by using BioDrop μLITE (BioDrop, UK). RIN value was determined using the 2100 BioAnalyzer (Agilent Technologies, CA, USA). A total of 1 µg of RNA was reverse-transcribed to cDNA using a High-Capacity cDNA Reverse Transcription Kit with RNase Inhibitor (Applied Biosystems^TM^, Thermo Fisher Scientific) according to the manufacturer’s instructions. cDNA samples were stored at −20 °C until further use.

### Nanolitre high-throughput qPCR

The expression of 18 genes (Table 1) in MSCs was evaluated by real-time PCR. The selection of those genes was mainly based in previous transcriptomic studies of cows treated with rbST and anabolic agents^26,27,31,32^. Of these 18 genes, three were used as endogenous controls to calculate the relative expression of the other 15 candidate genes. Gene expression profiling was carried out with a TaqMan^®^ OpenArray^®^ system (Applied Biosystems^TM^, Thermo Fisher Scientific) as described in a previous work^30^. This is a nanolitre high-throughput real-time PCR platform where 3,072 reactions are performed at the same time in the same OpenArray^®^ plate, and the primers and TaqMan^®^ probes are preloaded in the plates by the company. A plate design of 18 assays in triplicate and 56 samples was chosen. Real-time PCR reactions were performed according to the TaqMan^®^ OpenArray^®^ protocol. Briefly, in a 384-well plate, 1.2 µL of each cDNA sample was mixed with 3.8 µL of TaqMan^®^ OpenArray^®^ Real-Time PCR Master Mix (Applied Biosystems^TM^, Thermo Fisher Scientific). The PCR reaction mixtures were loaded automatically into the OpenArray^®^ plates using an OpenArray^®^ AccuFill™ System (Applied Biosystems^TM^, Thermo Fisher Scientific). The following real-time PCR protocol was used: 40 cycles at 95 °C for 15 s, and 60 °C for 1 min.

**Table 1:** List of genes selected to evaluate their expression in somatic cells.

Gene symbol	Gene name	NCBI Accession number of context sequence	ThermoFihser Assay ID*	Amplicon size (nucleotides)
IGF1	Insulin like growth factor 1	NM_001077828.1	bt03252281_m1	65
IGF1-R	Insulin like growth factor 1 receptor	NM_001244612.1	bt03649217_m1	70
IGFBP3	Insulin like growth factor binding protein 3	NM_174556.1	bt03223809_m1	77
IGFBP5	Insulin like growth factor binding protein 5	NM_001105327.2	bt03258785-g1	62
IL1β	Interleukin 1 beta	NM_174093.1	bt03212745_m1	129
TNF	Tumor necrosis factor	NM_173966.3	bt03259156_m1	69
LTF	Lactotransferrin	NM_180998.2	bt03217382_m1	95
COL3A1	Collagen type III alpha 1 chain	NM_001076831.1	bt03249914_m1	88
TPD52L2	Tumor protein D52 like 2	NM_001034615.2	bt03227133_m1	78
ESR2	Estrogen receptor 2	NM_174051.3	bt03259198_m1	73
CTNNAL1	Catenin alpha like 1	NM_001191534.1	bt04308229_m1	76
SIRT2	Sirtuin 2	NM_001113531.1	bt03258971_m1	59
CCND1	Cyclin D1	NM_001046273.2	bt03235030_m1	72
MFGE8	Milk fat globule-EGF factor 8 protein	NM_176610.1	bt03216856_m1	88
EEF1G	Eukaryotic translation elongation factor 1 gamma	NM_001040487.2	bt03229629_g1	74
**Reference genes**				
UXT	Ubiquitously expressed prefoldin like chaperone	NM_001037471.2	bt03229278_m1	89
RPS9	Ribosomal protein S9	NM_001101152.2	bt03272016_m1	65
GPAM	Glycerol-3-phosphate acyltransferase, mitochondrial	NM_001012282.1	bt03210379_m1	97

*Primers and probes used in this study are proprietary information of ThermoFisher Scientific. Information about amplicon size, context sequence and assay location can be consulted by introducing the assay ID on www.thermofisher.com webpage.

### Data analysis and statistics

#### RT-PCR data analysis

As described in a previous work^30^, the LinRegPCR software was used to analyse the raw real-time PCR data^33,34^ and calculate PCR efficiency. LinRegPCR imports non-baseline-corrected data and performs a baseline correction on each sample. Then, a window of linearity is determined, and linear regression analysis is used to determine the PCR efficiency per sample from the slope of the regression line. The mean PCR efficiency of each amplicon tested and the Cq value per sample were used to calculate a starting concentration (N_0_) per sample, expressed in arbitrary fluorescence units. After that, Factor Correction qPCR software was used to remove multiplicative between-session variation in experiments^35^. A session factor is used to correct the observed data and it can be calculated from a matrix of between-session ratios or estimated using a maximum likelihood approach. Corrected values are obtained by dividing the observed values by the session factor. Finally, the gene expression ratio was calculated by dividing the N_0_ of the target gene by the N_0_ of the geometric mean of the three reference genes.

Genes UXT, RPS9 and GPAM were used as reference genes and those genes were validated by using the BestKeeper® tool described by Pfaffl *et al*.^36^ for determination of housekeeping stable genes. As BestKeeper© Software is limited to 100 data form the same gene at the same time, we used the formulae described in the paper to calculate the stability of the genes.

#### Univariate statistical analysis

Statistical analyses were performed using the IBM SPSS Statistics software package for windows (SPSS Inc., Chicago, USA). Test T was used to determine significant differences between groups. The nonparametric Mann–Whitney U test was used when data was no normally distributed. A one-way ANOVA approach was applied for comparisons of more than two groups. Normal distribution was tested using Kolmogórov-Smirnov and Levene to assess the equality of variances.

#### Multivariate statistical analysis

Multivariate statistical analyses were carried out using SIMCA-P + 12.0 (Umetrics AB, Sweden). Firstly, gene expression data of nine genes measured in one hundred and ninety MSC samples from rbST-treated and control animals (IGF1R, CCND1, TNF, IL1β, SIRT2, EEFG1, MFEG8, LTF, TDP52L2) were logarithmically transformed and scaled according to the Pareto method. The other six target genes (IGF-1R, IGFBP3, IGFBP5, COL3A1, ESR2, CTNNAL1) were not included in multivariate statistical analyses because their expression was not detected or it was only detected in some samples. Principal component analysis (PCA) was applied for a non-supervised visualization of the transcriptomic profiles and to detect extreme observations/samples in the MSCs dataset. Outliers were selected from the PCA using Hotelling’s T2 and distance-to-model in X space tools. Accordingly, extreme samples were removed from the data matrix before any further processing; MSCs samples with missing values in their transcriptomic profile (applied criteria for inclusion: 0% missing observations) were also excluded. A supervised method, i.e. orthogonal projections to latent structures discriminant analysis (OPLS-DA), was secondly used adding information on class membership of the samples coded as Y variable (control samples or rbST samples). The quality of the OPLS-DA model was assessed by analysis of variance testing of cross-validated predictive residuals (CV-ANOVA), and its R^2^ and Q^2^ values. Lastly, to determine the variables (genes) more affected by rbST administration, and hence more appropriated to discriminate treated animals, the S-plot of the OPLS-DA model was used.

## Results and Discussion

### Effect of rbST on milk yield

Table 2 shows a summary of milk yield for the rbST and control groups expressed as mean and standard deviation of milk production (Kg per day) at each treatment cycle, as well as production difference between both groups (% of extra milk produced by rbST group in comparison to control animals). From cycle 0 to cycle 3, milk production seems to increase in both groups, possibly indicating that animals had not yet reached their peak of lactation. Although the first dose of rbST was administered around 2 months post-partum, as recommended by the manufacturer, it is important to note that this recommendation is based on mathematical models that locate the milk production peak at 60–90 days postpartum. However, these models are only theoretical approaches and days in milk (DIM) at the milk peak have a wide variation between herds and cows, being conditioned, amongst other factors, by welfare and feeding^37^. From cycle 4 to the end of the study, a decrease in milk production of control cows could be glimpsed while the rbST-treated animals seemed to maintain their production relatively constant on 30–35 kg average of milk per day, showing an obvious decreasing tendency only when the hormonal treatment was stopped (cycles 14, 15 and 16). At this point, the production differences observed between groups returned to levels very close to those observed at cycle 0. It is remarkable the fact that milk production was significantly higher in the rbST group than in the control during the whole study, except during cycle 6 when these animals were producing only 5% more than control subjects (Table 2). On the basis of the biweekly administration pattern of rbST recommended by the manufacturer, on day 70 the group of treated animals should have received a new dose of recombinant growth hormone. However, that dose was not applied so as to be able to evaluate if the rbST group could recover the pre-dose transcriptional profile after 28 days without hormonal administration (Fig. 1). Possibly due to this lack of hormone, the statistically significant differences existing in milk production between the two groups disappeared, highlighting the effects of rbST on milk production rates (Table 2). At day 84, rbST was administered again and the significant differences in milk production between the two groups reappeared (24%). A maximum difference of 43% more milk in treated animals with respect to control cows was observed at cycle 12, both related to a maintained production due to rbST and a natural decreasing pattern in control females. Curiously, these significant differences were maintained until the end of the study, even almost two months after the last dose (12^th^ dose, day 168) was administered (Table 2).

**Table 2:** Milk yield in treated (N = 6) and control group (N = 3) along the study.

	Milk yield (mean kg/day)			Days of study	Months in lactation (mean)	
	rbST (N = 6)	Control (N = 3)	Production difference (%)		rbST	Control
Cycle 0	40.41 ± 6.21**	33.94 ± 12.43	19.28 ± 4.96	−18 to 0	2.0	2.5
Cycle 1	40.53 ± 6.68**	35.43 ± 9.85	14.97 ± 7.14	1 to14	2.5	3.0
Cycle 2	42.22 ± 6.25**	37.38 ± 8.87	13.05 ± 4.99	15 to 28	3.0	3.5
Cycle 3	42.31 ± 5.84**	37.87 ± 8.01	11.80 ± 4.58	29 to 42	3.5	4.0
Cycle 4	38.49 ± 8.18*	35.00 ± 7.08	10.15 ± 6.45	43 to 57	4.0	4.5
Cycle 5	37.21 ± 8.27*	34.67 ± 6.65	7.38 ± 4.51	58 to 70	4.5	5.0
Cycle 6	35.00 ± 6.89	33.24 ± 4.73	5.44 ± 6.64	71 to 84	5.0	5.5
Cycle 7	35.09 ± 9.20***	28.43 ± 6.78	24.06 ± 8.99	85 to 98	5.5	6.0
Cycle 8	32.88 ± 7.92***	27.46 ± 4.59	19.89 ± 7.11	99 to 112	6.0	6.5
Cycle 9	36.13 ± 7.39***	29.18 ± 3.30	24.10 ± 6.04	113 to 127	6.5	7.0
Cycle 10	37.14 ± 6.29***	28.39 ± 2.83	30.99 ± 4.11	128 to 140	7.0	7.5
Cycle 11	34.70 ± 6.07***	27.42 ± 2.47	26.70 ± 7.35	141 to 154	7.5	8.0
Cycle 12	32.06 ± 5.50***	22.60 ± 6.86	43.26 ± 15.02	155 to 168	8.0	8.5
Cycle 13	32.31 ± 7.17***	24.75 ± 4.96	30.52 ± 10.56	169 to 182	8.5	9.0
Cycle 14	28.38 ± 6.55**	24.10 ± 5.39	18.22 ± 5.93	183 to 196	9.5	10.0
Cycle 15	29.82 ± 4.90***	25.91 ± 3.80	15.67 ± 9.77	196 to 209	10	10.5
Cycle 16	28.37 ± 4.85***	24.12 ± 5.50	13.03 ± 6.94	209 to 222	10.5	11

Data was divided in cycles based in rbST administration cycles of two weeks. The mean milk production of each cycle was calculated. The day 0 of study is the day of the first rbST dose administration. Cycle 6, 14, 15, 16 has no administration of rbST and the last day of the cycle correspond with the last sampling for transcriptomic assays. The test-T was used to compare the milk yield in each cycle between treated and control group.

### RNA isolation from bovine somatic cells

MSCs are easy-to-collect source of RNA for gene expression studies, as these cells can be isolated from raw milk following a very simple protocol. Besides, sample collection is cheap and non-invasive, since milk can be collected in the milking room without direct contact with the animal and therefore without causing stress to it which could alter the results obtained. In this study, the rbST treatment had no significant effect (*P* = 0.099) on the quantity of RNA isolated from MSCs, with a mean concentration of 120.69 ± 68.31 µg mL^−1^ in samples from the rbST group and 132.36 ± 68.86 µg mL^−1^ in samples from the control group. The mean A260/280 ratio value was 1.766 ± 0.095. It is possible that a purification step with spin columns increased the A260/280 ratio and therefore the purity of the RNA samples. Although the inclusion of this step increases the sample price in routine analysis, it allows obtaining samples with higher purity. The mean RIN value observed for the samples analysed was 6.88 ± 0.82 Some factors could affect to the integrity of the samples. The samples used in this study were bovine somatic cells isolated from milk. Milk is characterized by a complex microbiota and RNases derived from that microbiota could be responsible of a lower RNA integrity. In addition, some of somatic cells present in milk could be partially degraded. These facts were also observed in a study that used bovine vaginal smear for transcriptomics studies with the aim to find biomarkers to trace the misuse of anabolic agents^38^. Also, the process of milking, milk collection and transportation to the lab could, despite being carried out with the as swiftly as possible, affect to the RNA integrity.

### Effect of rbST on somatotropic axis genes

A set of 18 genes (Table 1) were selected for designing the OpenArray^®^ plates used in this study. This selection was based on the potential of these genes to obtain a characteristic rbST transcriptomic signature that could be used as a standard to control the ab(use) of rbST in dairy cattle. Previous gene expression studies with rbST were designed as single-dose and single sampling studies in post-mortem mammary tissue^27^ or multi-dose (five doses) studies with blood and muscle sampling *in vivo*^31^. Those methods are considered invasive, relatively expensive and therefore impractical for control purposes on dairy farms. In the case of somatotropin, the control must be performed *in vivo*, since the ultimate goal is to avoid the entry of rbST-milk into the dairy market.

In this study, the expression of IGF-1 was only detected at all the sample points in one cow (cow 7) treated with rbST and it was not detectable in the MSCs of control group. The fact that it was only possible to detect the IGF-1 target at all the sample points in one cow may indicate the existence of different local mammary gland responses to exogenous rbST among individuals. Most IGF-1 molecules found in the circulation are bound with high affinity to one or more of the six known IGF-1 binding proteins (IGFBP-1–6) that modulate the bioavailability of IGF-1 in target tissues^39^. Previous studies have observed that rbST strongly influences the upregulation of IGFBP-5 in skeletal muscle of cattle^31^ and downregulates IGFBP-3 in mammary tissue^32^. However, in this study, transcripts of neither IGFBP-3 nor IGFBP-5 were detected at any time point. This result could be due to MSCs being composed mainly of leukocytes^40^ in which the repressive effect of IGFBP may not be as important as in mammary tissue. Therefore, both IGF-1 and IGF-1 binding proteins are discarded as potential markers in somatic cells in this study.

**Figure 2:**
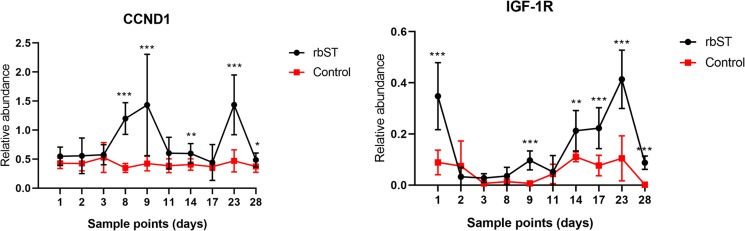
Relative abundance of IGF-1R and CCND-1 in the first and second cycles of rbST administration in control group (N = 3) and treated group (N = 6). The nonparametric Mann–Whitney U test was used to compare the milk yield in each cycle between treated and control group. Asterisks represent statistically significant differences between treated group and both control group. *(*p < *0.05), **(*p* < 0.01), ***(*p* < 0.001).

Another key component of the somatotropic axis is the IGF-1 receptor (IGF-1R) which is the primary signalling receptor for IGF-1 that mediates most of its biological effects. On day 1 the rbST group (0.348 ± 0.131) had a significantly (*P* < 0.001) higher relative abundance of IGF-1R than the control group (0.0898 ± 0.0486). Different studies that evaluated the concentration of rbST in blood after administration observed that the higher concentrations of this recombinant hormone were found after rbST administration^14,15^. Therefore, the significant differences observed in treated group could be due as a response to the IGF-1 synthesized as response the higher concentrations of rbST in treated cows after first dose administration. In Fig. 2, it can be observed that the relative abundance of IGF-1R for the two first cycles of rbST administration was also significantly higher in the rbST group than in the control group in many sample points. Curiously, on day 84 of the study, the relative abundance of IGF-1R was similar in the rbST group (0.070 ± 0.057) and in the control group (0.110 ± 0.030). This day coincided with the sixth administration of rbST, and between this dose and the fifth dose there was a gap of 28 days instead of 14 days. Therefore, it is possible that the rbST group recovered the physiological level of IGF-1R after several days without rbST, showing even withdrawal effects of down-regulation on IGF-1R transcription. These results indicate that IGF-1R is a possible good candidate for inclusion in a panel of genes to detect the use of rbST in dairy farms. Although other authors did not find a significant effect of rbST on IGF-1R levels in skeletal muscle^31^, in this study a clear response was observed in the levels of this receptor transcript in MSCs. In this sense, leukocytes, neutrophils and monocytes can produce growth hormone and IGF-1 and express their receptors that indicate that IGF-1/IGF-1R signalling pathways might exert regulatory functions on the immune system including immune cell proliferation^41,42^. The difference between the two studies could be due to the different matrices used. MSCs could possibly respond better to the higher levels of circulating IGF-1 and increase the number of receptors for this molecule in their membrane

### Effect of rbST on immune system-related genes

**Figure 3:**
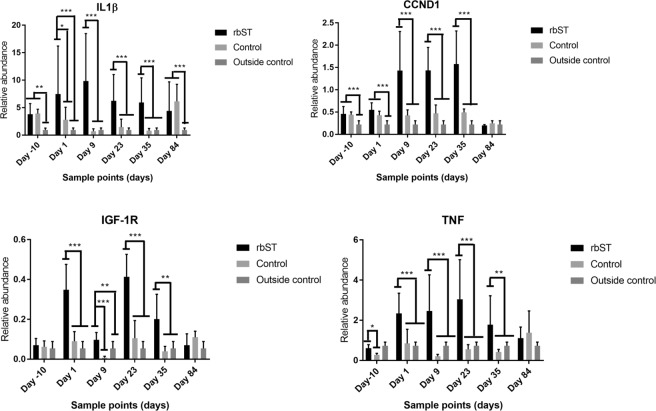
Relative abundance of CCND1, IGF-1R, IL1β and TNF transcription of rbST treated group (N = 6) and control group (N = 3) on days -10, 1, 9, 23, 35, 84. Samples for a single day of outside control cows (N = 3) were added. A one-way ANOVA approach was applied for comparisons of more than two groups. Asterisks represent statistically significant differences between treated group and both control group. *(*p < *0.05), **(*p* < 0.01), *** (*p* < 0.001).

Tumour necrosis factor (TNF) and interleukin-1β (IL-1β) are two cytokines closely related to the immune system^43^. Due presumably to their relationship, it was possible to identify similar trends in the relative abundance of TNF and IL-1β transcripts throughout the study. The rbST administration provoked a significant increase in the relative abundance of TNF and IL-1β transcripts. It is worth mentioning that on days 9, 23 and 35, the relative abundances of TNF and IL-1β were significantly higher in the rbST group in comparison to the control group (Fig. 3). These days correspond to the second week of rbST cycles, approximately 7–9 days after injections of Lactotropina®. Therefore, it is possible that the transcriptomic changes caused by rbST in these two cytokines are more evident in the second week of an rbST cycle. Curiously enough, on day 84 (28 days after the 5^th^ rbST administration), there was no significant differences between groups (Fig. 3). In this context, other previous studies have used powerful transcriptomic technologies to detect the use of anabolic agents in cattle^26,38^. These studies also observed an increased transcription of IL-1β, one of them significantly^26^, and this is very interesting because the referred study used blood cells which composition is very similar to that of MSCs, as they have a high proportion of white blood cells. However, the previously mentioned studies did not find an effect of anabolic agents on TNF, while the present research found a strong influence of rbST administration on the relative abundance of TNF. With this regard, it has been reported that the exogenous administration of rbST during lactation can enhance the immune response in cows^44^. Actually, milk somatic cell counts increased earlier and faster in cows suffering from coliforme mastitis when rbST was administered^45^. Growth hormone and its recombinant version show the ability to modulate the inflammatory reaction and neutrophil defense of the bovine lactating mammary gland in health and diseased cows^45^. This could explain the upregulation of TNF and IL-1β in rbST treated cows.

In screening studies by using transcriptomics it is very difficult to find a specific gene that could be used as very trusty gene. It should be noted that both IL-1β and TNF can be upregulated in other processes as sub-clinical mastitis^46,47^. Different studies carried out in the United States have observed that large farms are more likely to adopt rbST, suggesting not only a potential farm-size component of rbST use and profitability but also an operator (age and education) component^48,49^. Recombinant somatotropin has been frequently reported as a management-intensive technology, associated with the use of other productivity-oriented technologies and management practices that are characteristic of larger farms, being less frequent among grazers^49,50^. In this sense, subclinical mastitis causes a reduction in milk production in affected cows^51^. Therefore, it should be interesting to combine the data of transcriptomic assays with the milk production data. Also it could be accompanied by a milk microbiological assay of suspected cows to detect the principal pathogens associated with subclinical mastitis. But what really increases the potential for discrimination is the inclusion of more genes in the panel.

### Effect of rbST on cell cycle, proliferation, differentiation and adhesion

It is know that rbST increases milk synthesis by increasing the turnover (proliferation/apoptosis) and activity of mammary epithelial cells, indicating that rbST influences metabolic pathways that regulate cell turnover/cycle and metabolism^52^. The cell cycle is controlled by cyclins and cyclin-dependent kinases. Of those, cyclin D (endoded by the CCND1 gene) coordinates cell cycle progression through extracellular stimulation (e.g., growth factor, nutrient availability) and drives G1 to S phase progression resulting in cell mitosis. However, most adult cells are maintained in a quiescent state known as G0 phase, a resting state, and they can re-enter the cell cycle in G1 phase under appropriate mitogenic stimuli^53^. In this study, it was observed that rbST treatment strongly influences CCND1 transcription. Before the first dose of rbST, there were no significant differences between the control and rbST groups (Fig. 3). However, after the first dose, it was possible to observe significant differences between the two groups at different sample points. The relative abundance of CCND1 was significantly higher in the rbST group than in the control group in the two first cycles of rbST treatment (Fig. 2). This effect seems to be particularly evident 9 days after rbST administration, similarly to TNF and IL-1β transcripts. Thus, on days 9, 23 and 35 of the study, the relative abundance of CCND1 was significantly higher in the rbST group than in the control group (Fig. 3). Finally, on day 84 (28 days after the 5^th^ rbST administration), there were no significant differences between groups (Fig. 3) as in the case of the cytokines. Also, on the last day (219) of sampling (51 days after the last rbST doses), there were no significant differences (*P* > 0.05) between the rbST group (0.433 ± 0.141) and the control group (0.430 ± 0.178). Therefore, the exogenous administration of rbST causes an overexpression of CCND1. This can result in the activation of cell in G0 phase, and in the particular case of mammary tissue, an increase in milk production through increasing the number of alveolar cells of mammary gland^54^.

**Figure 4:**
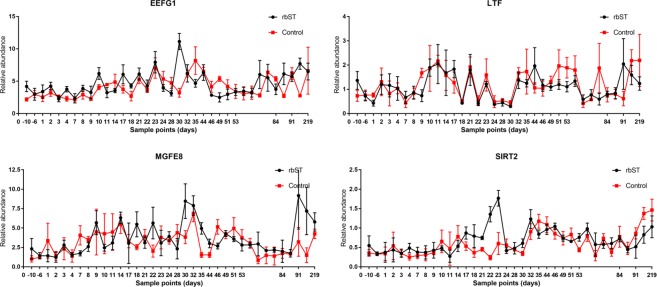
Evolution of the relative abundance of some target genes during all the sample points of the study in rbST treated group (N = 6) and control group (N = 3).

Other genes related to cell cycle are tumour protein D52-like 2 (TPD52L2) and sirtuin 2 (SIRT2). In a manner similar to that observed for CCND1, TNF and IL-1β, it was possible to find a significantly higher (*P* < 0.001) relative abundance of SIRT2 transcripts in the rbST group (1.517 ± 0.199) than in the control group (0.506 ± 0.250) on day 23 of the study, but in this case just during the second cycle of rbST administration (Fig. 4). The increased levels of CCND1 and SIRT2 transcripts after the second administration of Lactotropina® in the treated group could be the result of an effect of exogenous rbST on cell cycle. The circulation of this peptide hormone in the organism would result in the activation of the cell cycle in the target cells. However, in the other rbST cycles, the relationship between dose administration and changes in the relative abundance of SIRT2 transcripts was not as evident as in the second dose. In addition to the foregoing, it is remarkable that the treated group presented a significantly higher relative abundance of TPD52L2 transcripts (*P* < 0.05) during 4 days after the first rbST administration. Another component implicated in cell metabolism by its relevant role in translation is eukaryotic elongation factor 1 gamma (EEF1G).

In this study, the relative abundance of EEF1G transcripts did not follow an obvious tendency related to rbST administration. However, on days 17 and 30 (3 and 2 days after the second and third rbST doses, respectively), the relative abundance of EEF1G transcripts was significantly higher in the rbST group. A previous study^27^ concluded that rbST treatment increases the levels of EEF1G transcripts in mammary tissue but it only used one sample point 6 days after somatotropin administration. Therefore, it is not possible to directly compare those results with the results obtained in this study, in which 36 sample points were used for transcriptomic assays over the course of 8 months. Another gene included in this study is milk fat globule-EGF factor 8 protein (MFGE8). In Fig. 4, it is possible to observe that the relative abundance of MFGE8 in the rbST group had great variability between sampling days. In particular, McCoard *et al*.^27^ observed that 6 days after rbST administration, the relative abundance of MFGE8 in bovine mammary tissue was higher. However, as mentioned before, that study included only one data point after a single rbST dose. The present long-term multi-dose experiment has demonstrated that transcription patterns in bovine animals treated with rbST have great variability over time. For example, data obtained for CCND1 showed that the effect of rbST on the transcription of some genes increases with the number of doses. Although it is possible that rbST influences the transcription of the MFGE8 gene, obtained results showed both up- and down-regulation. Therefore, MFGE8 cannot be suggested as an ideal candidate for tracing rbST (ab)use in cattle, as it was not possible to observe a clear tendency in its transcription. Another gene related to cell adherence evaluated in this study is catenin alpha-like 1 (CTNNAL1). However, it was not possible to find significant differences in its transcription as a result of rbST administration.

### Other genes

Lactoferrin (LTF) is an iron-binding glycoprotein belonging to the transferrin family^55^. Figure 4 shows the evolution of LTF gene relative abundances at all sample points evaluated in this study. Although upregulation of LTF was observed in previous studies with anabolic substances in cattle^38^, it was not possible to establish a clear tendency for LTF in relation to rbST treatment. For example, on days 9 and 53, the relative abundance of LTF considerable lower in rbST-treated animals. However, on day 91, the relative abundance was considerable higher in that group. It is necessary to discuss the results obtained for the collagen type III α 1 (COL3A1) gene. A previous study concluded that treatments with rbST in dairy cattle cause upregulation of the COL3A1 gene in mammary tissue 6 days after rbST administration^27^. However, in the present work it was not possible to detect transcripts of the COL3A1 gene. Also ESR2 transcription was not detected in this study.

### A panel of genes to monitor rbST (ab)use in dairy cattle

The final aim of this work was to propose a routine panel of genes whose combined transcription pattern would allow the development of a screening method to control the misuse of rbST in dairy farms via MSCs. Unlike other previous studies^27,31,32^, this work analysed the transcription patterns of rbST-related genes in a 8-month real-conditions experiment including 12 cycles of rbST administration and control animals. This approach permitted more accurate data to be obtained in order to differentiate between rbST-treated cows and control cows.

**Figure 5:**
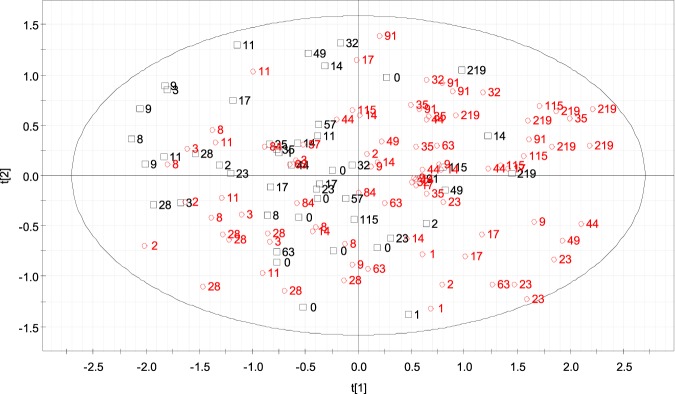
PCA plot built using full transcriptomic profiles of milk somatic cells (MSCs), in which a discrimination between cows treated with rbST (N = 6, red circles) and control animals (N = 3; black boxes) can be glimpsed. MSC samples are labelled according to day of experiment, being day 0 the day of the first rbST dose in treated animals.

Multivariate statistical analysis was run to elucidate modifications in MSCs transcription patterns as a consequence of rbST administration. For this purpose, only the transcript of the nine genes that were successfully measured over the whole experiment (IGF1R, CCND1, TNF, IL1β, SIRT2, EEFG1, MFEG8, LTF, TDP52L2) were used, excluding those genes that only expressed in very few cases/samples. The main goal of PCA analysis is to identify global patterns in data, detecting the correlation between different variables, i.e. in this particular case, transcriptions of different genes. Projection of the samples into the new multidimensional space of the principal components (PCs) would potentially allow a differentiation between rbST and control groups, highlighting also those genes with a greater ability as biomarkers of the treatment. In the score plot of the PCA analysis shown in Fig. 5, a grouping tendency could be glimpsed in the two groups of MSC samples, with rbST-treated animals identified as red circles and control animals as black boxes, and labelled according to the experimental day. Various samples of rbST group appeared mixed with controls on the left side of the plot but, curiously enough, the majority of them correspond to a day of somatotropin administration (days 14, 28, 57 or 84) or to the first 2–3 days after a dose (days 2, 3, 17, 44 or 115), in which an evident transcriptomic disturbance was not detected. On the contrary, very few control animals could be classified as treated cows on the PCA, and their situation is explained by extreme values in particular poorly discriminative genes such as LTF, MFGE8 or SIRT2.

**Figure 6:**
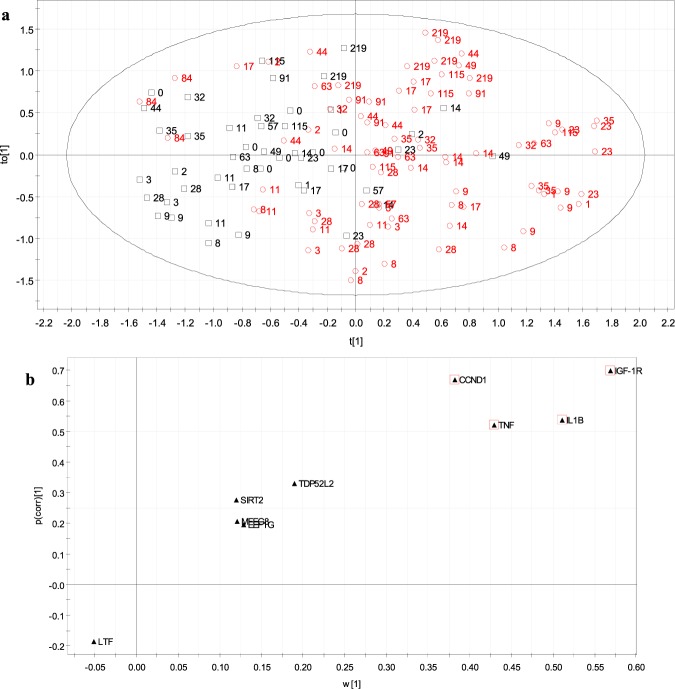
OPLS-DA scatter plot (**a**) constructed using trasncriptomic profiles from milk somatic cells, showing a discrimination between control (N = 3; black circles) and rbST (N = 6; red dots) animals, labelled according to the experimental day (being day 0 the day of 1st dose in rbST group). S-plot (**b**) associated to the OPLS discriminant analysis, highlighting the genes more affected by rbST on the upper right corner of the plot, and hence with a higher discriminative power.

The transcriptomic profiles were also subjected to an OPLS-DA, from which potential discriminative genes were also pointed out. In Fig. 6a, the visual representation of the multivariate model is shown, with MSC samples from control animals plotted as black squares and samples from rbST group as red circles. In a similar way to PCA, several rbST samples are projected among control samples on the left side of the ellipse. However, these samples were collected when the transcriptomic disturbance caused by rbST had disappeared (or had not appeared yet), so strictly, they could be classified as controls in that specific moment. Conversely, very few control samples were misclassified as rbST. When no test set is available as is this case here, the cross-validation method is the main strategy to assess the quality of a model. Results of the cross-validation procedure are summarized by the value of different quality parameters. In this study, the OPLS multivariate model showed the following characteristics: R^2^(X) = 0.6, R^2^(Y) = 0.4 and Q^2^ = 0.3. The model explains 60% of the variation in x-space and 40% of the variation in y-space, and goodness of prediction is 30%. These values indicate a relatively good description of the data by the model and average predictability^34^. The p-value calculated from the CV-ANOVA was 3.01 × 10^−7^, suggesting the existence of significant differences between the two classes of the model. The fact that this model was built with a panel of genes with different discriminating ability should not be ignored, as it affects the discriminating power of the OPLS. Besides, those experimental days with very little or no rbST-effect on their transcripts are definitely biasing the model since in the practice they belong to one group but act as the other. In future multivariate screening models, a panel of selected highly discriminative genes must be used, and preferably, “known” and “unknown” samples to be projected in the multivariate model will belong to similar populations (age, breed, etc.) and lactation moments, in an attempt to overcome inter-individual animal variability^35^. In this kind of approaches, it is also important that the predictive population is larger than the predicted samples. Finally, the reasons for discrimination on the OPLS-DA were investigated in the corresponding S-plot (Fig. 6b), which revealed the contribution of each variable (gene transcription) to the separation of the two sample classes. As commented in previous sections, the most discriminant transcription in MSC was found in CCND1, IGF-1R, TNF and IL-1β genes. It appears clear that rbST treatments trigger an upregulation of those genes and their usein future monitoring panels is a promising option for routine control in dairy.

As in a routine control to detect illicit use of rbST with this gene panel, there would be no information about when cows have been treated (in others words, it is a blind assay), ideally every day during lactation should be sampled/controlled. However, from a practical point of view, this suggestion sounds a bit unrealistic. What it is proposed is a random collection of milk once per week, alternating week days. As somatotropin is administered on a regular basis throughout lactation (starting 80–100 days postpartum, bi-weekly), in all lactating cows of the farm, with a good sampling plan, at some point rbST should be detected in the farm. The transcriptomic data obtained from the samples collected have to be compared with a transcriptomic dataset from a control population. Those samples that presented higher values of genes proposed in this paper should be considered as suspicious. The second step should be investigating the particular situation of each “positive” cow (mastitis, pregnancy, etc.), and, if required, continue to confirmatory analysis. It is important to highlight also the high-throughput capacities of this real time PCR approach, making feasible the sampling plan.

## Conclusion

This multi-dose longitudinal study with 12 rbST treatments and 36 sample points in real dairy farm conditions has allowed establishing a trustworthy transcription profile for both rbST-treated and controlling groups. These data in combination with multivariate statistical analysis showed that the transcription of CCND1, IGF-1R, IL-1β and TNF genes in MSC could be used as promising markers in a panel designed for screening of rbST misuse on dairy farms. Even though the implementation of this new approach is very challenging and sophisticated for real routine testing, it can be considered of valuable scientific interest and a starter point for future studies on the field.

## Data Availability

The datasets generated during and/or analysed during the current study are available from the corresponding author on reasonable request.
